# Relevance similarity: an alternative means to monitor information retrieval systems

**DOI:** 10.1186/1742-5581-2-6

**Published:** 2005-07-20

**Authors:** Peng Dong, Marie Loh, Adrian Mondry

**Affiliations:** 1 Medical Statistics and Epidemiology Group, Bioinformatics Institute, BMRC, A*STAR, Singapore

## Abstract

**Background:**

Relevance assessment is a major problem in the evaluation of information retrieval systems. The work presented here introduces a new parameter, "Relevance Similarity", for the measurement of the variation of relevance assessment. In a situation where individual assessment can be compared with a gold standard, this parameter is used to study the effect of such variation on the performance of a medical information retrieval system. In such a setting, *Relevance Similarity *is the ratio of assessors who rank a given document same as the gold standard over the total number of assessors in the group.

**Methods:**

The study was carried out on a collection of Critically Appraised Topics (CATs). Twelve volunteers were divided into two groups of people according to their domain knowledge. They assessed the relevance of retrieved topics obtained by querying a meta-search engine with ten keywords related to medical science. Their assessments were compared to the gold standard assessment, and *Relevance Similarities *were calculated as the ratio of positive concordance with the gold standard for each topic.

**Results:**

The similarity comparison among groups showed that a higher degree of agreements exists among evaluators with more subject knowledge. The performance of the retrieval system was not significantly different as a result of the variations in relevance assessment in this particular query set.

**Conclusion:**

In assessment situations where evaluators can be compared to a gold standard, *Relevance Similarity *provides an alternative evaluation technique to the commonly used kappa scores, which may give paradoxically low scores in highly biased situations such as document repositories containing large quantities of relevant data.

## Background

The advent of the Internet has changed the way both professionals and consumers look for health information [1]. Abbott [2] found that the existing general public search engines have a high penetration into even restricted-access data repositories, yielding quality information alternative to traditional primary sources. Recently, Google has launched a beta-version of its *Google Scholar *search engine, Nature Publishing Group has changed its search engine to allow deep penetration, and Elsevier has created another specialised search engine for scientific literature, *Scopus*, which comes with a cost [3]. All of these widen the general public's access to high-quality health information. But Peterson [1] showed that the generally low skill level for search strategies that most customers have could lead to retrieval of inadequate information, which raises anxiety and decreases compliance. In response to this, Curro [4] has suggested a simple methodology to assess the quality of medical information retrieved on the Internet, but the impact of this strategy remains to be seen. In the meantime, the medical professional is certainly better advised to look for information that has appraised content. Such sources include online repositories of Critically Appraised Topics (CATs). CATs are short summaries of current medical literature addressing specific clinical questions and are frequently used by clinicians who try to implement principles of Evidence Based Medicine (EBM) [5]. Although some CAT libraries exist, a peer-to-peer sharing network as proposed by Castro [6] is not yet available. CAT Crawler [7], an online search engine, provides access to a number of public online CAT repositories and is the focus of the present study on retrieval quality.

Two commonly used evaluation parameters are recall and precision [8]. The former measures the comprehensiveness of a search and the latter measures the accuracy of a search. Relevance is the key concept in the calculation of recall and precision but poses problems of multidimensionality and of dynamic quality. Schamber [9] has emphasized that relevance assessment differs between judges and for the same judge at different times or in different environments. Barry [10] and Schamber [11] have studied the factors affecting relevance assessments. Both studies have agreed that relevance assessments depend on evaluators' perceptions of the problem situation and the information environment, and the perceptions encompass many other factors beyond information content when they make the relevance assessment [12]. Only a few studies have directly addressed the effect of the variation in relevance assessments on the evaluation of information retrieval systems [13-17]. All studies varied relevance assessments with evaluators from different domain knowledge background. All of them concluded that variation in relevance assessments among judges has no significant effect on measures of retrieval effectiveness. However, Harter [18] has questioned this conclusion because none of these studies employs real users who approach the system for information need, although some of them tried to simulate this condition. He also highlighted the need to develop measurement instruments that are sensitive to variations in relevance assessments. A common statistical method used in this context is the kappa score, which, in principle, is a contingency table based method that can eliminate chance concordance from the assessment. However, modern search engines usually have filter systems [3], which lead to a selection bias towards relevant documents. Feinstein et al [19] observed that in situations with high imbalance, the paradox of high agreement but low kappa scores can arise. Better filters create more bias, thus increasing the tendency to find such paradox results. In such a situation, a performance assessment based on kappa scores may become meaningless.

The work presented here introduces a new parameter, *Relevance Similarity*, to address this problem. Based on this measurement parameter, the effect of the inter-evaluator variation of relevance assessment on the evaluation of the information retrieval performance was studied. The experiment was carried out on a collection of CATs. Two groups of evaluators participated in the relevance assessments on a set of retrieved topics from the medical meta-search engine, CAT Crawler.

## Methods

**Figure 1:**
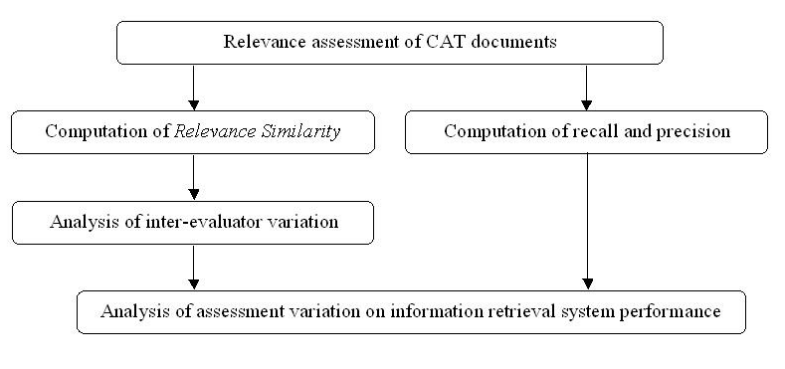
Workflow for analysing the effect of the inter-evaluator variation on CAT Crawler information retrieval system.

The retrieval system used in the study is the CAT Crawler meta-search engine. In a very brief summary, CAT Crawler can be described as a one-stop search engine for CATs stored over numerous online repositories. It has its own search engine, which allows the user to do a specific search rather than simply browse the repositories' contents. The CAT Crawler's standard setting has been shown to yield search results of equal quantity and enhanced quality compared to the original search engines available at some of the repositories [20]. The detailed structural design of CAT Crawler [7] has been described previously. The workflow of the CAT Crawler's evaluation is summarized in Figure 1.

### Relevance assessment of CATs in the test document set

Ten keywords (Table 1) related to medicine were chosen as the test seed and submitted to the search engine. All together 132 CAT links were retrieved and then evaluated for their relevance by 13 people, who were categorized into three groups according to their level of training regarding medical knowledge. Among them, one physician represents medical professionals and is considered as the gold standard for the evaluation, the six evaluators in Group A were trained in biology or medicine, while the six evaluators in Group B had no medical or biological background. For the sake of this exercise, the physician's evaluation of the relevance of each topic was taken as the gold standard or 'true' relevance of each retrieval result.

**Table 1:** CAT Link retrieval details. The numbers indicate how many documents were retrieved by the CAT Crawler meta-search engine.

**Keyword**	**Number of retrieved links**
Appendicitis	8
Colic	9
Intubation	22
Ketoacidosis	2
Octreotide	3
Palsy	10
Prophylaxis	30
Sleep	16
Tape	3
Ultrasound	29
	132

### Computation of *Relevance *Similarity

For each retrieved CAT, the evaluation by every participant in Group A and B was compared with the gold standard set by the medical professional. The *Relevance Similarity *is defined as:



*Relevance Similarity *was computed for each of 132 retrieved links. To compare the relevance assessment between Group A and B, a Chi-square test on the contingency table was carried out on all calculated *Relevance Similarity *values using the statistics software SPSS 11.0 (SPSS Inc., Chicago, IL, USA). In addition, kappa scores within evaluators of Group A and B were calculated respectively.

### Computation of recall and precision

In this study, the retrieval system performance is qualified by recall and precision. CATs containing a particular keyword are defined as "technically relevant" documents for that keyword [20]. In the first step, for each keyword, technically relevant documents were identified from the experimental document set and individual recall was computed for every evaluator accordingly. In the next step, the recall was averaged over all evaluators in a single group. Finally, the recall was averaged over the ten keyword queries. Following a similar process, the average precision was calculated.

### Computation of kappa score

To ensure the qualification of the physician as a gold standard, he re-evaluated the same document set a year after the initial assessment. A kappa score, observed agreement, positive and negative specific agreements between the two evaluations were calculated [21,22]. The inter-evaluator kappa scores within each group were computed for comparison.

## Results

### Analysis of the inter-evaluator variation

For each of the 132 retrieved links, *Relevance Similarity *was calculated for both Group A and B (Table 2). For instance, one CAT "*Plain Abdominal Radiographs of No Clinical Utility in Clinically Suspected Appendicitis*" was retrieved from  upon querying the meta-search engine with the keyword *Appendicitis*. The gold standard rated it as relevant; all six evaluators in Group A rated it as relevant too; whereas, one out of six evaluators in Group B rated it as irrelevant. The corresponding similarity for this particular CAT is computed as:

**Table 2:** Relevance Similarity for 132 retrieved CAT links. For each of the 132 documents retrieved by the CAT Crawler meta-search engine, Relevance Similarity (in %) was calculated for both Group A and B. *Link S/N *attribute is the serial number to each document.

**Link S/N**	**Group A (%)**	**Group B (%)**	**Link S/N**	**Group A (%)**	**Group B (%)**	**Link S/N**	**Group A (%)**	**Group B (%)**
1	100	83.33	45	100	100	89	66.67	33.33
2	83.33	66.67	46	50	50	90	50	83.33
3	100	100	47	50	33.33	91	50	66.67
4	100	100	48	50	66.67	92	66.67	83.33
5	100	100	49	100	100	93	33.33	83.33
6	100	100	50	100	100	94	50	50
7	100	100	51	100	100	95	33.33	66.67
8	100	100	52	66.67	50	96	100	100
9	100	100	53	33.33	16.67	97	100	100
10	100	100	54	100	100	98	100	100
11	0	0	55	100	100	99	100	66.67
12	100	100	56	100	100	100	66.67	66.67
13	100	100	57	100	100	101	100	100
14	100	100	58	100	100	102	66.67	83.33
15	100	100	59	100	100	103	100	100
16	100	100	60	100	100	104	83.33	100
17	83.33	83.33	61	66.67	33.33	105	100	100
18	100	100	62	83.33	33.33	106	100	100
19	66.67	83.33	63	16.67	83.33	107	100	100
20	66.67	50	64	50	83.33	108	100	100
21	50	66.67	65	100	100	109	100	100
22	33.33	66.67	66	66.67	83.33	110	83.33	66.67
23	100	100	67	0	33.33	111	83.33	83.33
24	50	50	68	50	50	112	83.33	100
25	50	66.67	69	0	16.67	113	83.33	33.33
26	83.33	50	70	66.67	50	114	100	100
27	66.67	100	71	83.33	66.67	115	100	100
28	50	66.67	72	100	83.33	116	100	100
29	100	100	73	50	83.33	117	83.33	66.67
30	50	50	74	100	66.67	118	83.33	66.67
31	100	100	75	100	83.33	119	83.33	66.67
32	83.33	83.33	76	100	100	120	83.33	66.67
33	100	66.67	77	100	83.33	121	100	66.67
34	100	83.33	78	66.67	50	122	100	83.33
35	100	100	79	83.33	83.33	123	100	83.33
36	100	100	80	100	100	124	100	100
37	33.33	16.67	81	83.33	66.67	125	100	100
38	83.33	66.67	82	100	66.67	126	66.67	66.67
39	66.67	50	83	66.67	33.33	127	100	100
40	50	50	84	83.33	66.67	128	83.33	33.33
41	100	100	85	83.33	100	129	33.33	50
42	100	100	86	100	100	130	66.67	83.33
43	16.67	50	87	83.33	100	131	83.33	66.67
44	100	100	88	33.33	50	132	83.33	100



**Figure 2:**
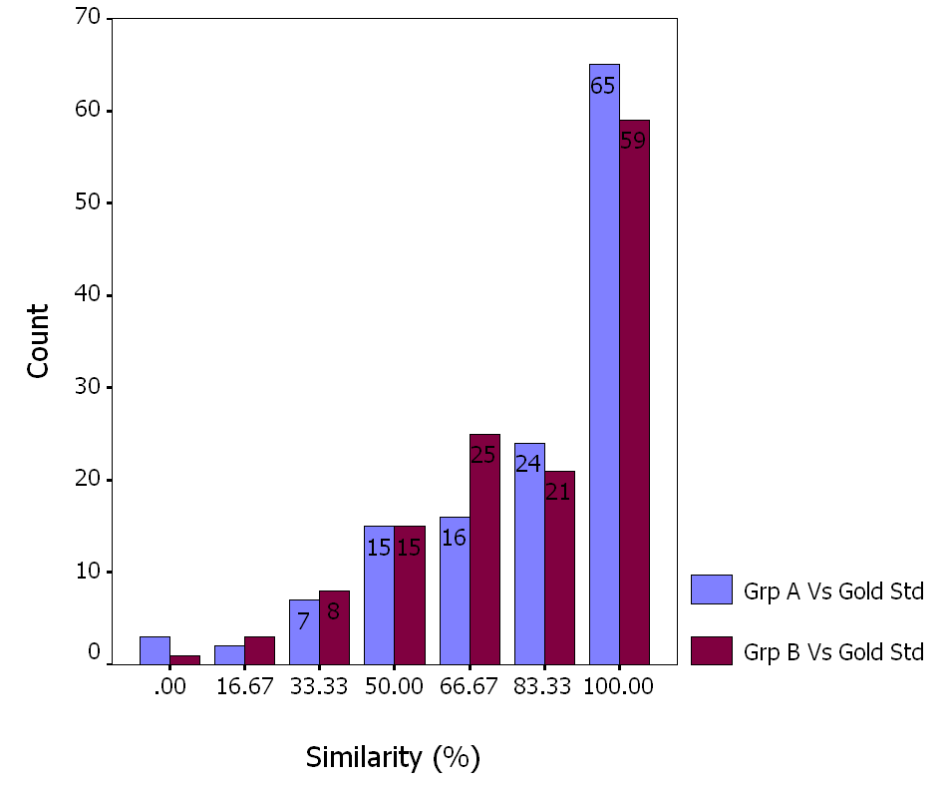
Frequency analysis of evaluation similarity of Group A and B versus the gold standard for all 132 CATs. Compared to the gold standard, the blue bar indicates the number of CATs evaluated by Group A at a different similarity level; the red bar indicates the number of CATs evaluated by Group B at a different similarity level.

Figure 2 shows the frequency analysis of *Relevance Similarity *for every retrieved CAT. Both Group A and B have evaluated around 90% of retrieved CATs with more than 50% similarity to the gold standard. The gold standard and the other two groups have made exactly the same relevance assessment on about half of the retrieved CATs. As shown in the last two columns of Figure 2, participators in Group A have evaluated 65 CATs (49%) with the same relevance as the gold standard; those in Group B have evaluated 59 CATs (45%) with the same relevance as the gold standard. The Chi-square test performed using SPSS between these two categories resulted in a *p-value *of 0.713.

### Evaluation of the retrieval system

**Figure 3:**
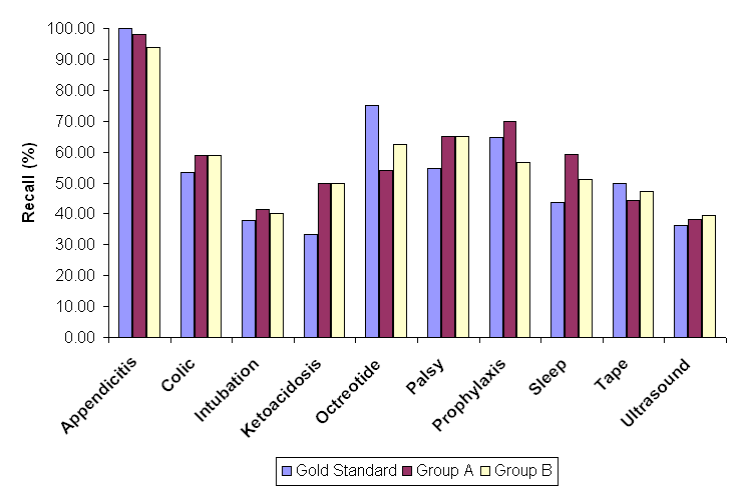
Recall comparison. The bars indicate each of the three groups' recall (in %) for the ten keywords.

**Figure 4:**
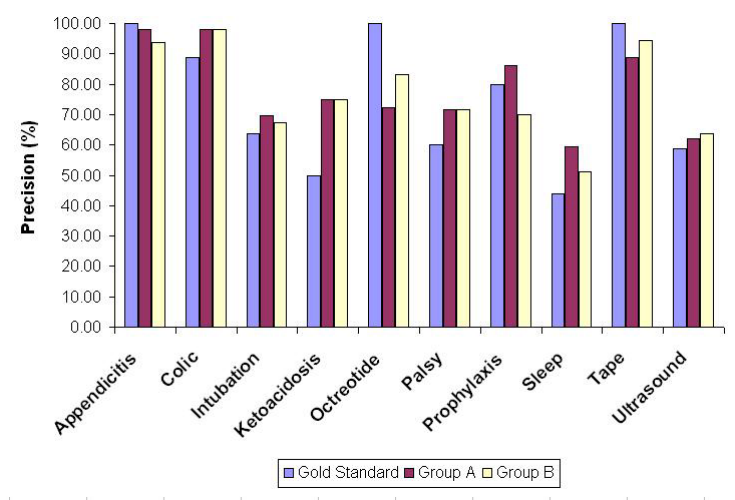
Precision comparison. The bars indicate each of the three groups' precision (in %) for the ten keywords.

Average recall and precision was computed for each keyword query and all numerical data are listed in Tables 4 and 5 respectively, while Figure 3 and 4 provide a more intuitive view of the recall and precision evaluation of retrieval.

**Table 4:** Average recall for the gold standard and the two groups of evaluators

	**Gold Standard**	**Group A**	**Group B**
**Appendicitis**	100.00	97.92	93.75
**Colic**	53.33	58.89	58.89
**Intubation**	37.84	41.44	40.09
**Ketoacidosis**	33.33	50.00	50.00
**Octreotide**	75.00	54.17	62.50
**Palsy**	54.55	65.15	65.15
**Prophylaxis**	64.86	69.82	56.76
**Sleep**	43.75	59.38	51.04
**Tape**	50.00	44.44	47.22
**Ultrasound**	36.17	38.30	39.36
**Average**	54.88	57.95	56.48

**Table 5:** Average precision for the gold standard and the two groups of evaluators

	**Gold Standard**	**Group A**	**Group B**
**Appendicitis**	100.00	97.92	93.75
**Colic**	88.89	98.15	98.15
**Intubation**	63.64	69.70	67.42
**Ketoacidosis**	50.00	75.00	75.00
**Octreotide**	100.00	72.22	83.33
**Palsy**	60.00	71.67	71.67
**Prophylaxis**	80.00	86.11	70.00
**Sleep**	43.75	59.38	51.04
**Tape**	100.00	88.89	94.44
**Ultrasound**	58.62	62.07	63.79
**Average**	74.49	78.11	76.86

### Kappa scores

The two evaluations of the document set carried out by the physician who served as the "gold standard" have a high concordance with a kappa score of 0.879. The inter-evaluator kappa scores ranged from 0.136 to 0.713 (0.387 ± 0.165) within Group A, and from -0.001 to 0.807 within Group B (0.357 ± 0.218) (Table 3).

**Table 3:** Kappa scores within Group A and Group B, de monstrating the paradoxically low kappa scores despite high agreement.

	**Group A**					**Group B**				
Evaluator	2	3	4	5	6	2	3	4	5	6

1	0.404	0.426	0.136	0.258	0.656	0.208	0.670	0.410	0.807	0.352
2		0.461	0.259	0.713	0.520		0.257	0.135	0.125	-0.001
3			0.180	0.438	0.439			0.440	0.643	0.353
4				0.241	0.270				0.370	0.250
5					0.404					0.330

## Discussion

Recall and precision remain standard evaluation parameters for the effectiveness evaluation of an information retrieval system. Both depend on the concept of relevance, i.e. the answer to the question whether the retrieved information is useful or not. A major problem lies in the fact that this answer may vary depending on multiple factors [9-11]. The perception of variance tempts one to assume that it must influence the assessment of retrieval efficiency, yet the small number of studies addressing this problem [13-17], including the one presented here, come to a different conclusion. This conclusion has been challenged [18], and the need to find measurement criteria for variance impact was recognized.

Three decades ago, Saracevic [23] has suggested to conduct more experiments on various systems with differently obtained assessments in the research of relevance variation. In contrast to previous studies, the present one investigates the effect of relevance assessment on the performance of a specialized retrieval system, developed specifically for physicians trying to implement EBM into daily routine. The test collection is a set holding around 1000 CATs. The variance of evaluator behavior is directly addressed by measuring *Relevance Similarity*. The concept of *Relevance Similarity *is strongly dependent on the knowledge of "true relevance".

It may be impossible to establish the true relevance of a given document. Whoever assesses a document may make an error. As soon as the document is assessed by another, the relevance may be attributed differently. For this reason, the "true relevance" is usually decided by expert committees, e.g. a group of specialists. Documents they assess in unison are assumed to be truly relevant or truly irrelevant; documents with variations in the assessment are either judged according to the majority's decision or following a brief decision rule.

In the present study, this problem was solved differently. According to the domain knowledge disparity between the evaluators, they could be categorized as: one medical professional, six life scientists and six IT scientists. From the training point of view, the physician is most closely related to the medical field and his judgement was therefore used as the gold standard or "true relevance". While one may (or may not) doubt his qualification to assign true relevance, his re-assessment of the same document set one year after his initial evaluation shows a good correlation. Using kappa statistics, a kappa score of 0.879 indicated an "excellent" concordance [24].

Kappa statistics are a standard measure of inter-evaluator agreement. In the present study, kappa scores for Group A evaluators ranged from 0.136 to 0.713, and from -0.001 to 0.807 for Group B (Table 3). Kappa statistics are based on the assumption that a "true" value is not known beforehand, and that a higher level of concordance signifies a higher probability to have a formed "truth". However, in situations where there is a strong bias towards either true or false positive, or true or false negative, high concordance can yield a low kappa score [19]. Positive and negative agreements have been suggested as an additional quality measurement in such cases. In the present study, we calculated positive and negative agreements [25] (P_pos_: 0.74–0.93; P_neg_: 0.15–0.82), but this does not give any additional information to that derived from kappa scores. While the calculation of kappa score does have its value, albeit not undisputed [19,25], to rely on this calculation misses a philosophical point: human evaluators may assess as true or false a statement that is not so for reasons that depend on external factors ("philosophies of life", political, theological etc.) and err with high concordance because they have concordance on the external factors. By assessing the documents using a gold standard considered to stand for the "true relevance", the method of *Relevance Similarity *overcomes this problem. Internal concordance of the gold standard evaluator is demonstrated by his excellent kappa score, and his study subject of medicine as opposed to life sciences/computer sciences qualifies him for this position.

With the physician as the gold standard, the *Relevance Similarity *for Groups A and B was computed for the analysis of these groups' agreement with the gold standard (Figure 2). For a high similarity level, Group A has more agreements with the gold standard than Group B. For example, for a relevance similarity level of 83.33%, Group A and the gold standard have evaluated 24 CATs with the same relevance. By comparison, Group B and the gold standard have an agreement over 21 CATs only. The same phenomenon occurs at a relevance similarity level of 100%. As the gold standard and Group A represent people with professional or some relevant medical domain knowledge, the result is consistent with what has been reported by Cuadra and Katter [26] and Rees and Schultz [27] that the agreement among evaluators with more subject knowledge is higher. On the other hand, a *p-value *of 0.713 shows there is no significant difference between the mean relevance assessment of Group A and B as compared to the gold standard.

Since the time of the Cranfield experiment [28], researchers have been aware of the difficulty of calculating the exact recall as this requires the true knowledge of the total number of relevant documents in the entire database. Even in the relatively small document repository used here that consists of around 1000 CATs in total, a visual control of all documents is unlikely to produce a reliable result in finding all files that contain the keywords, i.e. "technically relevant" documents. Using PERL scripts as described previously [20], this task is achieved reliably. The recall is computed accordingly.

The average recall and precision over all queries (Table 4 and 5) show that people with different domain knowledge have evaluated the retrieval system similarly. This supports the hypothesis of Lesk and Salton [13] that variations in relevance assessments do not cause substantial variations in retrieval performance. Their explanation is based on the fact that average recall and precision is obtained by averaging over many search requests. Concurring with this explanation, the average recall and precision for each keyword query in the present study (Table 4,5 and Figure 3,4) does vary between the gold standard, Group A and Group B in response to variations in relevance assessments for each keyword by different evaluators.

In this study, documents are judged for binary relevance, i.e. either relevant or irrelevant. Kekäläinen and Järvelin [29] have highlighted the multilevel phenomenon of relevance. The binary evaluation technique used in many studies is not able to represent the degree of relevance and hence leads to the difficulty of ranking a set of relevant documents. Recognizing the problem, many studies on information seeking and retrieval used multi-degree relevance assessments [30,31]. It would be worthwhile to consider the effect of multi-level relevance rating scales on the performance evaluation of the retrieval system.

## Conclusion

The present study directly addresses the question whether variability of relevance assessment has an impact on the evaluation of efficiency of a given information retrieval system. In the present setting, using a highly specialized search program exclusively targeting Critical Appraised Topics [7], the answer to that question is a clear "no" – the effectiveness of the CAT Crawler can be evaluated in an objective way.

To what extent the subject knowledge of the end-user influences his perception of relevance of the retrieved information is certainly important from an economic view, as it will have an impact on his usage patterns of information retrieval systems.

The results presented here demonstrate, however, that a safe evaluation of the retrieval quality of a given information retrieval system is indeed possible. While this does not allow for a qualitative control of the information contents on the plethora of websites dedicated to medical knowledge (or, in some cases, ignorance), the good news is that at least the technical quality of medical search engines can be evaluated.

## Competing interests

The author(s) declare that they have no competing interests.

## Authors' contributions

Author 1 (PD) participated in the design of the study, performed data analysis and drafted the manuscript. Author 2 (ML) has contributed on the statistical analysis of raw data. Author 3 (AM) participated in the design of the study and the drafting of the manuscript.
